# Cold burn injuries in the UK: the 11-year experience of a tertiary burns centre

**DOI:** 10.1186/s41038-016-0060-x

**Published:** 2016-11-11

**Authors:** Metin Nizamoglu, Alethea Tan, Tobias Vickers, Nicholas Segaren, David Barnes, Peter Dziewulski

**Affiliations:** 1 St Andrew Plastics and Burns Unit, Court Road, Chelmsford, CM1 7ET UK; 2 The University of Adelaide, Adelaide, South Australia 5005 Australia

**Keywords:** Cold burn, Guideline, Survey, Treatment

## Abstract

**Background:**

Guidance for the management of thermal injuries has evolved with improved understanding of burn pathophysiology. Guidance for the management of cold burn injuries is not widely available. The management of these burns differs from the standard management of thermal injuries. This study aimed to review the etiology and management of all cold burns presenting to a large regional burn centre in the UK and to provide a simplified management pathway for cold burns.

**Methods:**

An 11-year retrospective  analysis (1 January 2003–31 December 2014)  of all cold injuries presenting to a regional burns centre in the UK was conducted. Patient case notes were reviewed for injury mechanism, first aid administered, treatment outcomes and time to healing. An anonymized nationwide survey on aspects of management of cold burns was disseminated between 13 July 2015–5 October 2015 to British Association of Plastic Reconstructive and Aesthetic Surgeons (BAPRAS) and Plastic Surgery Trainees Association (PLASTA) members in the UK. Electronic searches of MEDLINE, EMBASE and the Cochrane Library were performed to identify relevant literature to provide evidence for a management pathway for cold burn injuries.

**Results:**

Twenty-three patients were identified. Age range was 8 months–69 years. Total body surface area (TBSA) burn ranged from 0.25 to 5 %. Twenty cases involved peripheral limbs. Seventeen (73.9 %)cases were accidental, with the remaining six (26.1 %) cases being deliberate self-inflicted injuries. Only eight patients received first aid. All except one patient were managed conservatively. One case required skin graft application due to delayed healing. We received 52 responses from a total of 200 questionaires. Ninety percent of responders think clearer guidelines should exist. We present a simplified management pathway based on evidence identified in our literature search.

**Conclusions:**

Cold burns are uncommon in comparison to other types of burn injuries. In the UK, a disproportionate number of cold burn injuries are deliberately self-inflicted, especially in the younger patient population. Our findings reflect a gap in clinical knowledge and experience. We proposed a simplified management pathway for managing cold burn injuries, consisting of adequate first aid using warm water, oral prostaglandin inhibitors, deroofing of blisters and topical antithromboxane therapy.

**Electronic supplementary material:**

The online version of this article (doi:10.1186/s41038-016-0060-x) contains supplementary material, which is available to authorized users.

## Background

Guidance for the management of thermal injuries has evolved accordingly with improved understanding of the pathophysiology of burn injuries. Extensive research has been conducted to improve management of thermal burns. Whist the national guidelines for burn resuscitation have been clearly outlined worldwide and taught through internationally recognized courses such as the Emergency Management of Severe Burns (EMSB) course, we feel that national guidance for the management of cold induced burn injuries is not widely available to clinicians. This may be partly because these injuries are relatively uncommon in comparison to other forms of burn injuries.

Frostbite or cold burn is the medical condition in which localized damage is caused to skin and other tissues due to freezing. Cold burns can occur through a variety of mechanisms ranging from prolonged exposure in a cold environment to the self-inflicted wounds from a seemingly benign aerosol can. The management of these burns differs from the standard management of thermal injuries. We performed a study to review our experience with the assessment and treatment of cold burn injuries. Additionally, a nationwide survey was disseminated to plastic surgeons in the UK to assess the current level of knowledge regarding managing cold burn injuries. We also conducted a review of current literature to provide a simplified management pathway of cutaneous cold injuries. Our recommendations are based on evidence graded using the American College of Chest Physicians (ACCP) classification criteria for grading evidence in clinical guidelines (see Table 1) [1]. This article focuses more on the cutaneous manifestation and treatment of cold injury rather than deep injury.

**Table 1:** ACCP classification criteria for grading evidence in clinical guideline

Grade	Description	Benefits vs. risks and burdens	Methodological quality of supporting evidence
1A	Strong recommendations, high-quality evidence	Benefits clearly outweigh risks and burdens or vice versa	RCTs without important limitations or overwhelming evidence from observational studies
1B	Strong recommendation, moderate-quality evidence	Benefits clearly outweigh risks and burdens or vise versa	RCTs with important limitations or exceptionally strong evidence from observational studies
1C	Strong recommendation, low-quality or very low-quality evidence	Benefits clearly outweigh risks and burdens or vise versa	Observational studies or case series
2A	Weak recommendation, high-quality evidence	Benefits closely balanced with risks and burdens	RCTs without important limitations or overwhelming evidence from observational studies
2B	Weak recommendation, moderate-quality evidence	Benefits closely balanced with risks and burdens	RCTs with important limitations or exceptionally strong evidence from observational studies
2C	Weak recommendation, low-quality or very low-quality evidence	Uncertainty in the estimates and burden; benefits, risks and burden may be closely balanced	Observational studies or case series

ACCP American College of Chest Physicians, RCTs randomized controlled trials

## Methods

An 11-year retrospective analysis (1 January 2003–31 December 2014) of all cold injuries presenting to a large regional burn centre in the UK was conducted. All patients admitted during this time period with diagnostic codes for ‘cold burns’, ‘frosbite’ and ‘cold injury’ were collated onto an Excel spreadsheet. Out of a total of 11,468 burns patients treated during the study period, 23 were identified as having cold injuries. The medical case notes were scrutinized for accuracy of documentation of first aid, patient demographics, management provided and defined outcomes, conservative or surgical management and healing times.

An online anonymized national survey on aspects of management of cold burns was disseminated nationwide between 13 July 2015–5 October 2015 to plastic surgery department staff and members of the British Association of Plastic Reconstructive and Aesthetic Surgeons (BAPRAS) and the Plastic Surgery Trainees Association (PLASTA). In total, 200 questionnaires were distributed. The survey questions consisted of a subjective perception of competence to treat cold-induced burns, whether specific training has been provided, the frequency of encountering cold burns, awareness of any topical therapies for cold burn injuries, the need for clearer guidance on the management of cold burns and lastly if further training in the management of this condition would be beneficial (Additional file 1). This piece of work has been registered with the hospital’s clinical governance department.

Electronic searches of MEDLINE, EMBASE and the Cochrane Library were undertaken in Jan 2016 using different combinations of the words "cold" "burns" "injury" "frostbite" "management" "review" and "treatment". Relevant articles were independently selected from titles and abstracts and then from the full text of the manuscript. Further articles that were missed by the search were identified by a manual search of the references of key articles.

## Results

### Our experience in the management of cold burns

**Fig 1:**
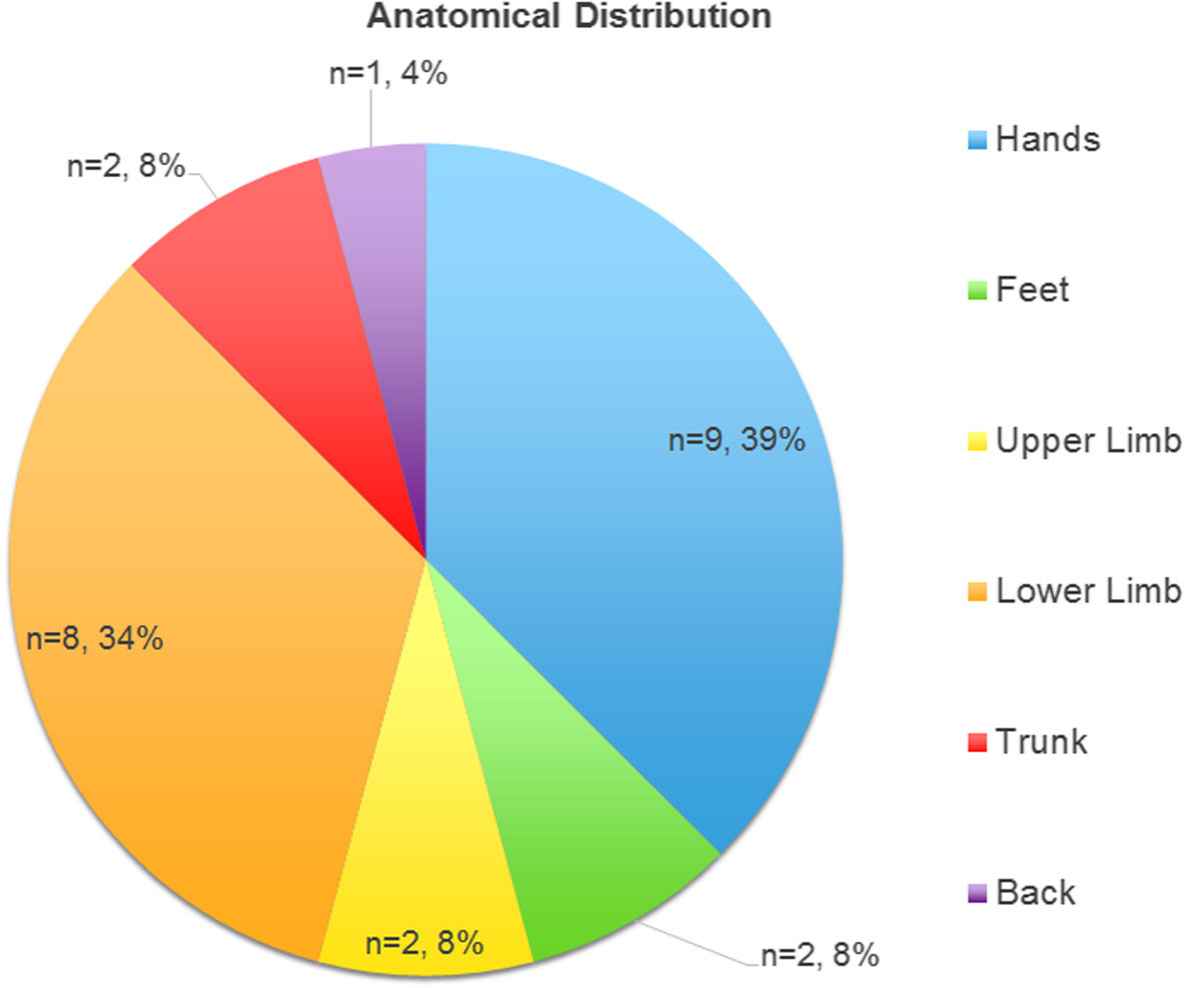
Anatomical location of cold burn injuries

Twenty-three patients were identified from the database. Patient details are summarized in Table 2. Mean age was 30 years (range 0.67–69 years). Mean total body surface area percentage (TBSA %) burn was 1.1 % (range 0.25–5 %). Twenty cases were partial-thickness injuries and three cases were full-thickness injuries. Seventeen (73.9 %) cases were accidental injuries and six 26.1 % cases were deliberate self-inflicted injuries. The full-thickness injuries in our study were all secondary to deliberate self-harm inflicted by aerosol exposure. The majority of the injuries affected the patient’s hands, followed by their lower limbs. The trunk was the least affected area. Anatomical distribution of injury is summarized in Fig. 1.

**Table 2:** Patient Demographics

Variables	Total, n (%)
Patients	23 (100 %)
Patient Age	
Median	30 years
Range	0.69 – 69 years
TBSA%	
Mean	1.1 %
Range	0.25 – 5 %
Depth	
Full thickness injury	3 (13.0 %)
Partial thickness injury	20 (87.0 %)
Cause	
Accidental	17 (73.9 %)
Deliberate self-harm	6 (26.1 %)
Mechanism	
Iatrogenic cryotherapy	3 (13.0 %)
Contact	5 (21.7 %)
Industrial	4 (17.4 %)
Deodorant	7 (30.4 %)
Dry Ice	1 (4.3 %)
Environmental	3 (13.0 %)
Management	
Conservative	22 (95.7 %)
Surgical	1 (4.4 %)
Healing Time	
Mean	19 d
Range	3-56 d

TBSA% total body surface area %

This most common mechanism was deodorant sprays, followed by direct prolonged contact with ice packs. Other less frequent causes were iatrogenic cryotherapy treatment for skin lesions, environmental exposure, industrial-related accidents and dry ice contact. Our results show that 8 (34.8 %) of patients received first aid, consisting of warm or tepid running water. Only 3 (13 %) of patients wore protective equipment consisting of one or more of the following; gloves, eye goggles, aprons and / or protective footwear. With regard to aerosol-inflicted injuries, one cases had duration of exposure recorded of 1 min. None had distance from spray nozzle or number of sprays documented. In the age group 15–25 years, 90 % of admissions were due to deliberate self-inflicted injuries using deodorant sprays. Only eight patients had first aid administered with either warm water or tepid water.

Mean healing time was 19 days (range 3–56 days). All of our patient cohort, particularly those exposed to the cold environment for prolonged periods of time suffered simple (grade 1) Cauchy classification frostbite injuries with no need for amputation of the digits [2]. All patients were managed conservatively initially. Only one case required wound excision and skin graft application after failing to heal by day 38. Following surgery, graft take was successful and complete wound closure was achieved on day 13 post-operation. This injury was sustained as a result of placement of an ice pack against the skin over the right thigh for several hours in order to alleviate musculoskeletal pain.

### Survey findings

We noted common themes of lack of structured clinical guidelines for management of these injuries and a discrepancy in the knowledge of how to manage cold burns. We feel this experience may be shared by other plastic surgeons. Among 200 questionuaires distributed, there were 52 (26 %) responses. Survey results are summarized in Table 3. Responders were from varying grades ranging from burn research nurse to consultant grade. Responses were obtained nationwide from the following regions: London, East of England, South West, Thames Valley, Midlands, Yorkshire, Northern England, Scotland, Wales and Northern Ireland. Only 37(72 %)  responders felt they were competent in managing cold burn injuries. Twenty-eight (54 %) responders had previously received training specific to the management of cold burn injuries. Interestingly, only 18 (35 %) of the responders confirmed a clinical guideline or protocol existed for cold burn management at their unit. The most widely cited source of guidance was the EMSB course, although other sources quoted included Scottish Intercollegiate Guidelines Network (SIGN), Advanced Trauma Life Support Course (ATLS) and local trust policy. Forty-six responders stated the number of cold burn cases presenting per annum to their unit was less than ten with the majority quoting this figure as <2 per year. Alarmingly, 71 % of responders were not aware of the availability of topical agents that can be used to prevent the damage caused by cold burns. Interestingly, 90 % responders felt there should be clearer guidelines on the management of cold burn injuries and 85 % stated they would benefit from further training on the management of cold burn injuries.

**Table 3:** Summary of survey

Variables	n(%)
Total questionnaires	200
Responder	52(26 %)
No response	148(74 %)
Grades	
Consultants	9 (17 %)
Registrars	36 (69 %)
House Officers	3 (6 %)
Nurses	2 (4 %)
Undisclosed	2 (4 %)
Competence managing injuries	
Felt competent	37 (71 %)
Felt incompetent	15 (29 %)
Use of clinical guidelines	
Yes	18 (35 %)
No	34 (65 %)
Type of guidelines used	
EMSB	40 (77 %)
Others (SIGN, ATLS, NICE, Local Trust)	12 (23 %)
Knowledge on available topical treatment	
Aware	15 (29 %)
Unaware	37 (71 %)
Opinion regarding need for further training	
Yes – Beneficial	44 (85 %)
No	8 (15 %)

*ATLS* Advanced Trauma Life Support Course, *EMSB* Emergency Management of Severe Burns, *SIGN* Scottish Intercollegiate Guidelines Network, *NICE* The National Institute for Health and Care Excellence

### A simplified management pathway for cold burns

The evidence base for the proposed management pathway was identified by our literature search outlined in the methods section (Table 4).

## Discussion

The pathophysiology of cold burn injuries differs to that of thermal injuries. Whilst the latter is more common and clear guidelines exist for its management, it is important for burn units to understand the underlying mechanism of cold burn injuries and offer appropriate treatment. The mechanism of peripheral cold injuries can be divided into direct effects on the cell and extracellular fluid and indirect effects disrupting the integrity of the circulation and function of organized tissue [3]. Cellular injury may be due to intracellular water crystallization, temperature-induced protein changes and membrane damage [4]. Slow cooling crystallizes extracellular water, which decreases interstitial water in the liquid phase and draws water out of cells. This effect alters intracellular electrolyte concentrations, which modifies cellular protein structure [5]. The cell content becomes hyperosmolar, and toxic concentrations of electrolytes may cause cell death [6]. Vasoconstriction, endothelial injury and thromboembolism contribute to vascular insufficiency and ischemia. Prostaglandins may play a role [7]. Vasoconstriction causes hypo-perfusion and stasis. Endothelial injury causes thrombosis and loss of vascular integrity. Thromboembolism, from stasis and endothelial injury, may also be promoted by haemoconcentration and hyperviscosity. Bleeding may be caused by cold-induced inhibition of coagulation cascade enzymes and platelet dysfunction [8, 9]. As the tissues thaw, oedema occurs because of the melting water crystals, cellular damage, loss of endothelial integrity and thrombosis. Haemorrhage can occur as well. Over time, necrosis becomes apparent.

In the initial treatment of a cold injury, non-adherent wet dressings should be removed. Local re-warming should begin only if the risk of refreezing has been eliminated. Thawing then refreezing results in more extensive injury (evidence grade 1B) [10, 11]. Rubbing affected areas worsens damage. In hospital, rapid rewarming of the extremity/area in a bath of hot water between 40 and 42 °C for 15–30 min may minimize tissue loss [12]. The Wilderness Medical Society and State of Alaska Cold Injury Guidelines recommend a temperature of 37–39 °C as this decreases the pain experienced by the patient (evidence grade 1B) [10, 11]. Hypothermia should be corrected bringing core temperature above 35 °C before warming (evidence grade 1C) [10, 13]. Analgesia and tetanus vaccine should be administered as required. Tetanus prophylaxis is indicated because frostbite injuries are considered tetanus-prone wounds [14]. Management of blisters is controversial. Some advocate their removal because of high concentrations of prostaglandin F2-alpha and thromboxane A2 in the exudate [15]. Both prostaglandin F2 and thromboxane A2 cause platelet aggregation and vasoconstriction. Therapy with anti-prostaglandin agents and thromboxane inhibitors has been shown in experimental and clinical studies to increase tissue survival.

It is recommended that clear blisters are drained by needle aspiration and haemorrhagic blisters left alone [10]. We suggest that all blisters are debrided as it assists with wound care and allows more accurate assessment of the depth of injury which therefore contributes to management decisions. In severe injuries, debriding of blisters may be best performed under a general anaesthetic (evidence grade 2C) [16]. The affected body part, especially limbs, should be elevated and splinted with a loose protective dressing (evidence grade 1C). Splinting and elevation of the affected limb may reduce oedema and promote tissue perfusion. These simple interventions can help minimize tissue damage and should be part of first aid management of cold burn injuries.

Heggers et al. recommended a therapeutic approach devised to prevent the progressive dermal ischemia of frostbite. The combination of the systemic prostaglandin inhibitor ibuprofen and the topical antithromboxane agent aloe vera was used to inhibit localized thromboxane production, which had been implicated as the cause of dermal ischemia [4]. Oral ibuprofen 12 mg/kg twice daily provides systemic anti-prostaglandin activity that limits inflammatory tissue damage. This dose can be increased to a maximum of 2400 mg/day. Alternatively, aspirin 300 mg can be given once a day (evidence grade 2C) [17]. Topical aloe vera cream or gel (an anti-prostaglandin agent) should be applied to the tissue before dressings are applied (evidence grade 2C) [10].

Severe frostbite can result in the loss of limbs and digits. The effect of tissue plasminogen activator and heparin in limb and digit preservation has been demonstrated by Twomey et al. [18]. Bruen et al. further confirmed the reduction of the incidence of amputation in frostbite injury with thrombolytic therapy [19]. Angiography after severe frostbite is a sensitive method of detecting impaired arterial blood flow and permits catheter-directed thrombolytic treatment. Improved perfusion after such treatments decreases late amputations following frostbite injury [20]. Prostacyclin analogue has also been demonstrated to reduce amputation rates in digits with severe frostbite (evidence grade 1B) [16, 21].

The difficulty in accurately determining the depth of tissue destruction has led to a conservative approach of care for local cold injuries. As a general rule, cold burn injuries are initially managed conservatively to allow the wound to demarcate, unless severe infection with sepsis develops [22]. Systemic antibiotics are required only in the presence of proven infection, trauma or cellulitis (evidence grade 1C). If the wound is deep dermal or full thickness and of significant size, early excision and grafting is advocated as with other thermal injuries [23]. However, from our experience, the size of these injuries are relatively small; therefore, even the full-thickness injuries from our cohort were managed conservatively due to the small surface area involved and the self-inflicted causation, making them poor surgical candidates for wound care compliance. Emergency surgery is occasionally required for patients with a frostbitten extremity. Post thaw reperfusion injury may lead to compartment syndrome which mandates fasciotomy (evidence grade 1C) [22, 24]. Open amputations are indicated in patients with persistent infection with sepsis that is refractory to debridement and antibiotics. Premature amputation increases morbidity and is likely to lead to poor subsequent function (evidence grade 1C). Most amputations can be performed once demarcation of ischaemic tissue has been well defined at 6–12 weeks post injury [17]. Negative pressure wound therapy (NPWT) can reduce healing time of amputation sites when left to heal by secondary intention [25].

Our experience demonstrates that cold burn injuries encompass a range of both young and elderly patient populations. The mechanisms of injury include environmental, accidental, deliberately self-inflicted and iatrogenic causes. The results demonstrate that less than half of our cohort received first aid following the injury. This may be due to cold injuries being less commonly encountered compared to other forms of burn and therefore a lack of practitioner familiarity with techniques used in treating these wounds. Six (26 %) case of all cold injuries were due to self-harm. This appears to be a disproportionately high percentage when compared to other mechanisms of burn injury. This may be due to the easily accessible nature and seemingly harmless deodorant that can be easily concealed and camouflaged as an accessory without suspicion of misuse. In the younger age group, almost all of the cases were due to deliberate self-harm. Aerosols used to inflict harm is a phenomenon well-documented in the literature. It is important to document distance from skin and duration of aerosol spray to skin as the greater the distance from the skin the slower the cooling rate [26].

From our experience, we have identified two common underlying aetiological factors consisting of psychiatric personality disorders and a perceived form of demonstrating courage when challenged by peers. Iatrogenic causes consisted of self-applied ice packs and cryotherapy for cutaneous lesions. All of our cold burn wounds were initially managed conservatively, with all but one healing without surgical intervention. Our data demonstrates conservative management resulted in healing of 93 % of our study population with a mean time of 19 days. This may demonstrate that partial thickness cold burn injuries have a propensity to heal following conservative management; however, this would be dependent on the size and depth of injury. This may reflect preservation of progenitor cells within the dermis following cold induced injury; however, more research would need to be performed to ascertain this with any degree of certainty.

In one study, human skin equivalents (HSEs) were used to reproduce burn and cold injury in vivo. They found novel similarities and differences involved in the closure of the two different types of wound in a fully defined, in vitro, HSEs wound healing model [27]. They found the rate of re-epithelialization was significantly slower after introduction of burns compared to cold injury. They also found that after burning, in contrast to cold injury, the basement membrane is damaged and has to be re-synthesized in order to permit re-epithelialization. Although morphological differences were observed between the two types of wound and no differences were observed in the profiles of the chemokines secreted. Our cohort of patients all had wounds less than or equal to 5 % TBSA with an average of 1 %; therefore, it can be argued that these wounds are relatively ‘small’ areas and would have healed by conservative measures eventually. Furthermore, healing time may have been significantly shorter if surgical intervention was performed for more cases and at an earlier stage. Prolonged conservative management may have been secondary to inexperience of the practitioner in managing these rare injuries or possibly the injuries themselves appearing to be deceptively more superficial than they were.

The survey results clearly demonstrated a gap in knowledge in the management of cold burn injuries. Majority of responders were not even aware of any topical agents that are available to prevent cold burn-related tissue damage. With a plethora of guidelines available for the management of burn injuries, surprisingly there is very little focus on the management of cold injuries. The guidelines quoted by our responders such as the EMSB and Scottish Intercollegiate Guidelines Network (SIGN), whilst providing guidance for burns in general (including electrical and chemical burns), do not offer specific guidance for the management of cold-induced burn injuries. The updated ATLS manual ninth edition currently includes a brief section outlining management recommendations [28]. We postulate that the lack of attention to these types of injuries may be due to its infrequent presentation. The survey results overwhelmingly demonstrated that there is a need for clearer management guidance for these injuries.

**Table 4:** A management pathway for cutaneous cold induced burns

Intervention (level of evidence)	Reference
1. First aid with warm water 37–39 °C for 30 min–1 h. (1B)	[10, 11]
2. Analgesia including ibuprofen (prostaglandin inhibitor) unless contraindicated. (2C)	[17]
3. Escharotomy ± fasciotomy if clinical suspicion of compartment syndrome. (1C)	[22, 24]
4. De-roofing of blisters (under general anaesthetic if indicated). (2C)	[16]
5. Aloe vera (antithromboxane) gel topically. (2C)	[10]
6. Elevation, splinting and barrier dressings. (2C)	[10]
7. Antibiotics if evidence of infection. (1C)	[22]
8. In severe frostbite wounds to extremities or digits, thrombolytic/prostacyclin therapy is to be considered. (1B)	[16, 19, 21]
9. Resurfacing of wound if indicated with skin graft ± skin substitute. (2C)	[23]

A limitation of this study was retrospective data collection based on documentation in the case notes. We could not prospectively assess the depth of burn or document the duration and distance of exposure to the cold stimulus accurately, a detail that is seldom documented in the medical notes. However, studies on the relationship between these factors and degree of tissue damage have been previously performed. Another limitation is the possibility of responder bias in any survey undertaken. However, we feel that the overwhelmingly one-sided view received by responders has given us an overall fair insight into the opinions on the knowledge deficit in cold burns management and the need for clear guidelines and training.

## Conclusions

Cold-induced burn injuries are uncommon in comparison to other types of burn injuries. Our findings have shown inadequate management of these injuries implicating a possible gap in knowledge and clinical experience in managing these rare conditions. This finding is also supported by our survey results. In the UK, a disproportionate number of cold burn injuries are deliberately self-inflicted, especially in the younger patient population. These patients should be managed in a multidisciplinary environment including clinical psychologists. Effective management of cold burn injuries starts with appropriate first aid and clear documentation of specific information unique to cold burns. A simplified management pathway highlighting key points for consideration at each stage (history taking, clinical evaluation, first aid, observation first 24 h, management options and follow-up care advice) will be a useful guide for clinicians when dealing with rare cases of cold burns. More research must be done on the physiology surrounding re-epithelialization and healing in the cold injury in contrast to a thermal burn to aid practitioners in managing this condition.

## Additional file

Additional file 1: Supplementary material: survey questions. (PNG 16 kb)

## Abbreviations

ACCP: American College of Chest Physicians
ATLS: Advanced Trauma Life Support Course
BAPRAS: British Association of Plastic Reconstructive and Aesthetic Surgeons
EMSB: Emergency Management of Severe Burns
HSE: Human Skin Equivalent
NICE: The National Institute for Health and Care Excellence
NPWT: Negative pressure wound therapy
PLASTA: Plastic Surgery Trainees Association
RCTs: Randomized controlled trials
SIGN: Scottish Intercollegiate Guidelines Network
TBSA: Total body surface area
