# Supplementary figures and images for: Cold burn injuries in the UK: the 11-year experience of a tertiary burns centre
Source: Burns Trauma. 2016 Nov 11;4:36. doi: 10.1186/s41038-016-0060-x (PMC5105282; doi:10.1186/s41038-016-0060-x)

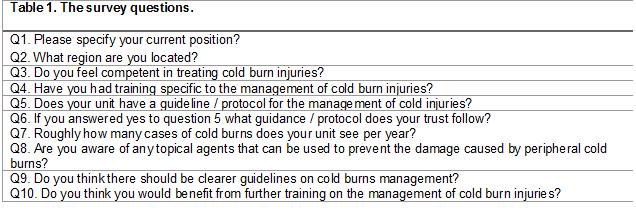

Supplement: Supplementary file 1 — Supplementary material: survey questions. (PNG 16 kb) [file 41038_2016_60_MOESM1_ESM.png]
